# Enhancing conjugation from *E. coli* to *Streptomyces coelicolor* by incorporating *traJ* into mobilizable plasmids

**DOI:** 10.1007/s00253-025-13662-4

**Published:** 2025-12-09

**Authors:** Paula Valdés-Chiara, Yago Concha, Sergio Alonso-Fernández, Angel Manteca, Gemma Fernández-García

**Affiliations:** Department of Functional Biology, Microbiology Area, IUOPA and ISPA, Faculty of Medicine, Universidad de Oviedo, c/ Julian Claveria 6, 33006 Oviedo, Spain

**Keywords:** *Streptomyces*, Conjugation, TraJ, Genetic manipulation

## Abstract

**Abstract:**

*Streptomyces* species are prolific producers of bioactive natural products, yet their genetic manipulation remains constrained by inefficient DNA delivery methods in many strains. Conjugation from methylation-deficient *Escherichia coli* has become the preferred approach for introducing plasmids into *Streptomyces*, relying on the presence of the *oriT* sequence within the mobilizable plasmid and the conjugation machinery (*tra* genes) encoded on the non-mobilizable helper plasmid pUZ8002. Among these, *traJ* encodes an essential component of the relaxosome. An additional copy of *traJ* is present downstream of *oriT* in some mobilizable plasmids, whereas many other commonly used plasmids lack *traJ*. Here, we investigated the impact of including *traJ* in mobilizable plasmids on conjugation efficiency by engineering two *oriT*-containing plasmids that initially lacked *traJ*: the ΦC31 integrative vector pRASK-SP44 and the non-replicative transposon delivery vector pHL734. We also examined the effect of introducing a second copy of *traJ* into the recombination-based chromosomal end-removal vector pCER. Incorporation of *traJ* into pRASK-SP44 and pHL734 resulted in tenfold and 100-fold increases in transconjugant numbers, respectively. Furthermore, introducing a second copy of *traJ* into pCER led to a fivefold improvement in plasmid transfer. Our data suggest that the inclusion of *traJ* improves transfer efficiency and may help overcome limiting steps in conjugation from *E. coli* to *Streptomyces*. Modulating the presence and copy number of *traJ* could represent a simple yet effective strategy to enhance genetic accessibility in *Streptomyces*. These findings have broad implications for the optimisation of genetic tools used in *Streptomyces* genome engineering and natural product discovery.

**Key points:**

• *traj in mobilizable plasmids enhances conjugation to S. coelicolor.*

• *traj increases plasmid transfer efficiency up to 100-fold in S. coelicolor.*

• *traj may aid development of genetic tools for genome engineering in Streptomyces.*

**Supplementary Information:**

The online version contains supplementary material available at 10.1007/s00253-025-13662-4.

## Introduction

*Streptomyces*, the major representative of the GC-rich Gram-positive Actinobacteria, is renowned for producing a wide array of natural products with valuable pharmacological, agricultural, and industrial applications (Donald et al. 2022; Kim et al. 2025). Their secondary metabolites exhibit diverse bioactivities, including antibiotic (e.g. teicoplanin, tetracycline), anticancer (e.g. doxorubicin, actinomycin D), immunosuppressive (e.g. rapamycin, sirolimus), and antiparasitic properties (e.g. ivermectin, selamectin) (Barreiro et al. 2024; Cerna-Chávez et al. 2024; Procopio et al. 2012; Rui et al. 2025). Indeed, *Streptomyces* species are responsible for producing over two-thirds of currently used antibiotics (Zong et al. 2022).

The biotechnological relevance of *Streptomyces* has driven the development of genetic tools to enable natural product discovery and metabolic engineering. However, DNA transfer is the essential first step in genetic manipulation and remains a major bottleneck in many *Streptomyces* strains (Krysenko 2025). Among the earliest DNA delivery methods was polyethylene glycol (PEG)-mediated transformation of protoplasts, following the pioneering work of Okanishi et al. (1974) on protoplast generation in *S. griseus*. Later, Hopwood and colleagues enabled efficient transformation of protoplasts from various *Streptomyces* strains using covalently closed circular plasmid DNA with PEG, osmotic stabilisers, and defined media (Bibb et al. 1978). Despite its utility, this method remains labour-intensive and strain-dependent due to variability in protoplast formation and regeneration (Du et al. 2012; Gomez-Escribano et al. 2025; Song et al. 2019; Zhang et al. 2019).

In the mid-1990s, electroporation emerged as a more rapid and reproducible alternative. Pigac and Schrempf (1995) reported efficient electroporation of *Streptomyces rimosus*, achieving transformation frequencies of up to 10⁶ transformants per microgram of plasmid DNA under optimised conditions. The method avoids protoplast generation and has since been adapted to various *Streptomyces* species, although the requirement for strain-specific optimisation of voltage, buffer composition, and cell wall weakening remains a limitation (Fan et al. 2013).

Conjugation from methylation-deficient *E. coli* into *Streptomyces* was first demonstrated in *S. coelicolor* using RP4-derived transfer functions (Bierman et al. 1992; Le et al. 2025; Mazodier et al. 1989). It has since become the preferred method due to its simplicity, efficiency, and ability to bypass restriction–modification barriers (Kieser 2000), with broad application in strain engineering and natural product discovery (Sun et al. 2014; Zhang et al. 2019). Nevertheless, DNA uptake remains challenging in some strains (Wu et al. 2025). Conjugative DNA transfer relies on the *oriT* sequence and a suite of *tra* genes, which encode a relaxase, a mating pair formation complex, and a type IV coupling protein (Llosa and de la Cruz 2005; Smillie et al. 2010). The relaxosome, formed at *oriT*, introduces a site-specific nick via the relaxase (Guzman-Herrador and Llosa 2019) and is bridged to the mating pair formation complex by the coupling protein (Cascales and Christie 2003). Proteins such as TraJ and TraK are essential for relaxosome formation and provide specificity to their native *oriT* (Li and Christie 2020; Pansegrau et al. 1988). *Streptomyces* plasmids mobilizable by conjugation carry *oriT* from the RK2 IncP plasmid, while the conjugation machinery is supplied *in trans* by the methylation-deficient *E. coli* strain ET12567, which harbours the non-mobilizable helper plasmid pUZ8002 encoding the necessary transfer machinery (Flett et al. 1997; Larcombe et al. 2024; Paget et al. 1999) but lacks a functional *oriT*. This setup bypasses restriction enzyme barriers and facilitates DNA delivery. Notably, the *traJ* gene in the parental RK2 conjugative plasmid is located near the *oriT* (Pansegrau et al. 1994), leading to its co-cloning in several widely used mobilizable plasmids such as ΦBT1-based integrative plasmids (pMS82) (Gregory et al. 2003), ΦC31 integrative plasmids (e.g. pSET152, pIJ10257) (Bibb et al. 1985; Combes et al. 2002), and multicopy expression vectors (e.g. piJ86) (https://actinobase.org/index.php?title=PIJ86). However, in other plasmids mobilizable by conjugation, only *oriT* was cloned. Examples include ΦBT1-based integrative plasmids (pRT802) (Gregory et al. 2003), φC31 integrative plasmids (pRAS) (Perez-Redondo et al. 2010), plasmids used for CRISPR-Cas9 mutagenesis (pCRISPomyces-2, pCRIDPR-Cas9) (Cobb et al. 2015; Tong et al. 2015), and plasmids designed for gene deletion selection (pIJ12742) (Fernández-Martínez and Bibb 2014).

In the present study, we systematically investigated the effect of incorporating the *traJ* gene into the mobilizable plasmids pRASK-SP44 (ΦC31-integrative) (Fernández-García et al. 2024) and pHL734 (non-replicative in *Streptomyces*, harbouring the mini-Tn5 transposon) (Xu et al. 2017), both of which lack *traJ*, as well as by introducing a second copy of *traJ* into the chromosomal-end-removing (pCER) mobilizable plasmid developed in our laboratory for homologous recombination-mediated deletion of chromosomal ends (unpublished). A substantial increase in the number of transconjugants was observed for all plasmids carrying either one or two copies of *traJ*, compared with the original plasmids lacking *traJ* or carrying only a single copy.

## Materials and methods

### Bacterial strains and growth conditions

*Escherichia coli* DH5α (Invitrogen) was used as a general host for cloning and plasmid propagation. Methylation-deficient *E. coli* ET12567 (*dam*−13::Tn9, *dcm*−6, *hsdM*, *hsdS*) (MacNeil et al. 1992), harbouring plasmid pUZ8002 (Larcombe et al. 2024), was used as the donor strain for conjugation experiments (Table 1). *E. coli* strains were cultured at 37 °C in liquid 2 × TY medium with shaking at 250 rpm, or on solid 2 × TY medium. Chloramphenicol (25 μg/ml), apramycin (100 μg/ml), hygromycin (100 μg/ml), nalidixic acid (25 μg/ml), and kanamycin (25 μg/ml) were used as selective antibiotics for *E.coli* when necessary.

**Table 1:** Bacterial strains, plasmids and primers used in this study

Strain	Description	Reference
*S.coelicolor* M145	SCP1^−^ SCP2^−^, reference strain	(Kieser 2000)
*E. coli DH5α*	*F-Φ80lacZΔM15 Δ(lacZYA-argF)U169 recA1 endA1 hsdR17 (rk-, mk* +*) poa supE44 thi-1 gyrA96 relA1λ-*	Invitrogen
*E. coli* ET12567/pUZ8002	*E. coli* ET12567 harbouring pUZ8002, a non-self-transmissible plasmid which can mobilize *oriT*-containing plasmids by conjugation	(Flett et al. 1997)
Plasmids	**Description**	**Reference**
pUC57-OriT TraJ	Cloning vector harbouring OriT, TraJ sequence; Kan^R^, Apra^R^	This study
pRAS	pRA^3^ vector modified by Antonio Rodríguez and Alberto-Sola-Landa	(Perez-Redondo et al. 2010)
pRASK-SP44	pRASK^4^ vector harbouring SP44 promoter*,* Kan^R^, Apra^R^	(Fernández-García et al. 2024)
pRASK-SP44-TraJ	pRASK^9^ vector harbouring SP44 promoter and TraJ sequence*,* Kan^R^, Apra^R^	This study
pHL734	Tn5-based transposon vector; Amp^R^ Apr^R^	(Xu et al. 2017)
pHL734-TraJ	pHL734 harbouring TraJ; Amp^R^ Apr^R^	This study
pCER	*Streptomyces* chromosomal-end-removing vector Kan^R^, Hygro^R^	Unpublished data
pCER-TraJ	Recombinant vector in *Streptomyces* harbouring TraJ*.* Kan^R^, Hygro^R^	This study
Primer	**Sequence**	**Reference**
pHL734-TraJ F	CAGTCGAAGCTCGACGGGTT	This study
pHL734-TraJ R	ATCTACACGACGGGGAGTCA	This study
pCER-2TraJ F	GTCCACGACCCTCCGTG	This study
pCER-2TraJ R	TCCTTGGTGTATCCAACGGC	This study
pRASK SP44-TraJ F	CGGAAGTGCTTGACATTGGG	This study
pRASK SP44-TraJ R	TATAGGGCAGCGCGCGTAA	This study
TnF	GCAGCTCCATCAGCAAAAGG	This study
TnR	GACGCTGCCGACGAAAAATC	This study
HygF	GGTACCAGTGAGCGTTTTTCAACCT	This study
HygR	AAAGACAATCCCCGATCCGCTC	This study
3798intF	CAGCTCGTCCTTGGTGTTCA	(Fernández-García et al. 2022)
3798intR	TCAGGTCCATGACGTTTCCC	(Fernández-García et al. 2022)

*Streptomyces coelicolor* M145 (Table 1) was grown at 30 °C on solid soy flour mannitol (SFM) medium for spore collection, on solid yeast extract–malt extract agar (GYM) medium (Novella et al. 1992) (5 g/L glucose, 4 g/L yeast extract, 5 g/L malt extract, 0.5 g/L MgSO₄·7H₂O, 20 g/L agar; supplemented post-autoclaving with sterile 0.5 g/L K₂HPO₄) for transconjugant growth, and in liquid R5A medium with shaking at 250 rpm to harvest mycelia for chromosomal DNA extraction. Apramycin (50 μg/ml), kanamycin (25 μg/ml), and hygromycin (200 μg/ml) were added as selective antibiotics for *Streptomyces* when required. *S. coelicolor* spores were harvested from 7-day-old SFM-grown cultures at 30 °C using Milli-Q water. Spore concentrations (spores/mL) were estimated by OD₆₀₀ using a calibration curve generated by correlating OD₆₀₀ readings with CFU. Aliquots were stored in 30% (v/v) glycerol at − 80 °C.

### Construction of plasmids harbouring traJ

**Fig. 1:**
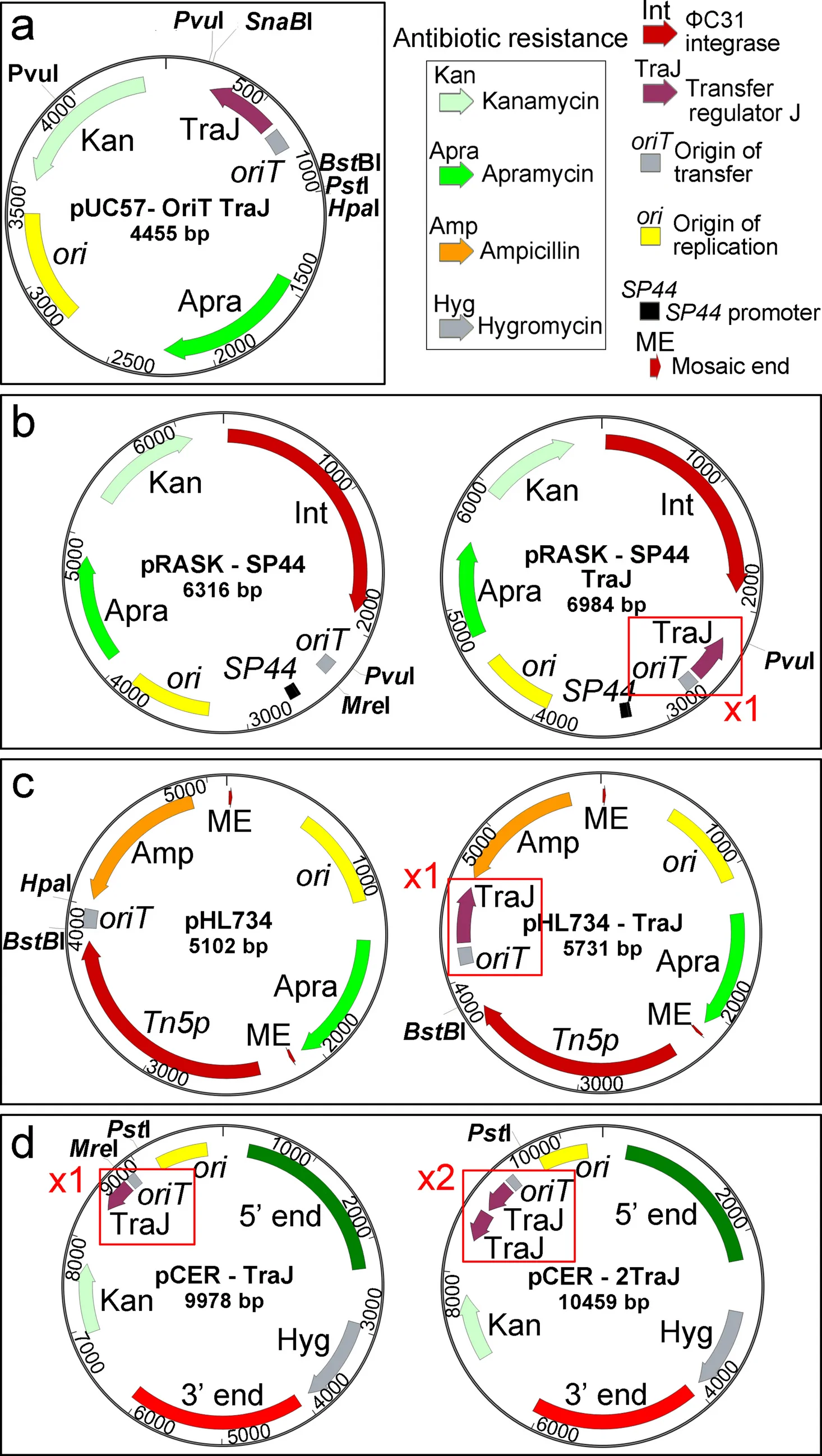
Construction and maps of the ΦC31 integrative plasmid pRASK-SP44 and the transposon delivery vector pHL734 harbouring *traJ*, and of the pCER chromosomal end-removal plasmid carrying two copies of *traJ*. **a** Map of the parental plasmid pUC57-oriT-*traJ*, from which the *oriT*-*traJ* fragment was extracted. **b** Maps of the pRASK-SP44 plasmid without *traJ* (left) and with *traJ* (right). **c** Maps of the pHL734 plasmid without *traJ* (left) and with *traJ* (right). **d** Maps of the pCER-*traJ* plasmid (harbouring one *traJ* copy, left) and pCER-2*traJ* (harbouring two *traJ* copies, right). See Methods for details of the cloning strategy

The plasmids used in this study are summarised in Table 1, and their maps are shown in Fig. 1. We began with the plasmid pUC57-OriT TraJ. The apramycin resistance gene together with the *oriT traJ* fragment was generated by gene synthesis and cloned into pUC57 by GeneCust (Boynes, France).

To construct pRASK-SP44 TraJ, a 6,163 bp fragment was generated by digesting pRASK-SP44 (Fernández-García et al. 2024) with *MreI* (Thermo), treating with S1 Nuclease (Thermo), and digesting with *Pvu*I (EurX). This was ligated to an 821 bp fragment obtained by digesting pUC57-OriT TraJ with *Hpa*I (EurX) and *Pvu*I (EurX).

To construct pHL734-TraJ, a 4,958 bp fragment was obtained by digesting pHL734 (Xu et al. 2017) with *Hpa*I (EurX) and *Bst*BI (Thermo). This fragment was ligated to a 771 bp insert generated by digesting pUC57-OriT TraJ with *Sna*BI (Thermo) and *BstBI* (Thermo).

To construct pCER-2TraJ, a 9,684 bp fragment was obtained by digesting pCER (unpublished) with *Mre*I (Thermo), treating it with S1 Nuclease (Thermo), and subsequently digesting it with *Pst*I (EurX). This fragment was ligated to a 783 bp insert obtained from pUC57-OriT TraJ digested with *Sna*BI (Thermo) and *Pst*I (EurX).

All ligation reactions were carried out in a final volume of 10 μL using T4 DNA Ligase (Invitrogen) and 5 × T4 DNA Ligase Buffer (Invitrogen) at a vector:insert ratio of 3:1. Ligation mixtures were incubated at 23 °C for 1 h or at 14 °C for 16 h. Correct assembly of the recombinant plasmids was verified by restriction digestion and Sanger sequencing, performed by Stabvida (Portugal), using plasmid-specific primers: pHL734-TraJ F/R, pCER-2TraJ F/R, and pRASK-SP44-TraJ F/R (Table 1).

### Solid-surface conjugation assay

Intergeneric conjugation between *E. coli* and *S. coelicolor* was performed as described by Kieser et al. (2000), with minor modifications. A volume containing 1.5 × 10⁸ frozen *S. coelicolor* spores was thawed and resuspended in 500 µL of R5A medium. The spore suspension was heat-treated at 50 °C for 10 min and then mixed with 500 µL of an overnight culture of the *E. coli* ET12567 donor strain (grown for 14 h in 2 × TY medium supplemented with the appropriate antibiotics) harbouring the mobilizable vector and the helper plasmid pUZ8002. The donor culture was prepared by harvesting 1 mL by centrifugation, washing three times with antibiotic-free 2 × TY medium, and resuspending in 500 µL of fresh 2 × TY. Donor cells were used in the 1 mL conjugation at final concentrations of 2.84 × 10⁸, 1.8 × 10⁹, and 1.32 × 10⁹ cells/mL for conjugations with pRASK-SP44, pHL734, and pCER-TraJ, respectively, with *S. coelicolor* at a final concentration of 3 × 10⁸ spores/mL. Concentrations of the *E. coli* ET12567 donor strain were measured just prior to mixing with *Streptomyces* spores by means of the optical density at 600 nm (OD₆₀₀). Serial dilution plating was also performed to verify these cell density estimations once *E. coli* colonies became visible the following day. As the *E. coli* ET12567 (*dam*⁻, *dcm*⁻) strain is particularly fragile, accurate estimation of colony-forming units is essential. We established a calibration curve for each strain to account for potential differences in growth fitness among the *E. coli* strains harbouring different plasmids. This was particularly necessary for the strain carrying pRASK-SP44, which showed approximately one order of magnitude fewer viable cells than the others after overnight culture. These differences in overnight cultures explain why different donor densities were used in the conjugation experiments.

The mixture of *Streptomyces* spores and *E. coli* donor cells was spread onto SFM plates supplemented with 10 mM MgCl₂ and incubated at 30 °C for 18 h. Following incubation, plates were overlaid with 1.5 mL of Milli-Q water containing 25 µg/mL nalidixic acid (to eliminate *E. coli*) and the appropriate selective antibiotic. Plates were then incubated at 30 °C for 7–10 days until transconjugants appeared.

Conjugation efficiency was calculated as the number of transconjugants per donor cell. Experiments were performed in triplicate, and results are reported as mean transfer frequencies ± standard deviation. Data normality was assessed using the Shapiro–Wilk test, and homogeneity of variance was evaluated using Levene’s test. Statistical significance was determined using one-sided t-tests, with *p*-values < 0.05, < 0.01, and < 0.001 considered significant.

### Analysis of the integration of ΦC31 pRASK-SP44, the mini-Tn5 transposon, or pCER in transconjugants

Three representative transconjugants from each conjugation experiment were streaked onto GYM medium supplemented with nalidixic acid and the appropriate selective antibiotic to obtain sufficient biomass for inoculation into liquid R5A medium. Mycelium from liquid cultures was harvested and genomic DNA was extracted using the salting-out method described by Kieser et al. (2000).

The presence of each plasmid in the transconjugants was confirmed by PCR. For pHL734 and pHL734-TraJ, a 951 bp fragment within the mini-Tn5 transposon was amplified using primers TnF/R (Table 1), confirming the transposon insertion. For pCER-TraJ and pCER-2TraJ, a 1,759 bp fragment within the *hygromycin* resistance gene was amplified using primers Hyg F/R (Table 1), confirming the presence of the plasmid. For pRASK-SP44 and pRASK-SP44-TraJ, a 981 bp fragment was amplified using primers 3798int F/R (Table 1). PCR reactions were performed in 20 μL volumes containing 1.25 U Taq DNA Polymerase (EurX), 10 × Pol Buffer B (EurX), 2 ng of template DNA, 0.2 mM of each dNTP, 0.5 μM of each primer, and 2 μL of DMSO. The PCR programme consisted of an initial denaturation at 95 °C for 5 min, followed by 30 cycles of 95 °C for 30 s, annealing at the appropriate temperature for 45 s, and extension at 72 °C for 1 min per kb of expected product size, with a final extension at 72 °C for 10 min.

## Results

### Construction of ΦC31 pRASK and Tn5-containing pHL734 plasmids harbouring traJ, and of the pCER plasmid carrying two copies of traJ

As introduced above, this study aimed to investigate the role of *traJ* in conjugation. The gene *traJ* is present in some plasmids mobilizable by conjugation but absent in others, and we examined how its presence affects the efficiency of conjugation from *E. coli* to *Streptomyces*. We selected three conjugation-mobilizable plasmids (i.e., carrying *oriT*) that are unable to replicate in *Streptomyces* and integrate into the chromosome via distinct mechanisms. These are: pRASK-SP44, a ΦC31 integrative plasmid (Fernández-García et al. 2024); pHL734, which harbours a mini-Tn5 transposon and enables random genomic integration (Xu et al. 2017); and the chromosomal-end-removing plasmid pCER, developed in our laboratory based on the method described by Volff et al. (1997). pCER contains homologous regions corresponding to the 5′ and 3′ ends of the chromosome, allowing recombination-mediated deletion of chromosomal ends and subsequent circularisation (unpublished). Both pRASK-SP44 and pHL734 contained *oriT* but lacked *traJ*, whereas pCER possessed both *oriT* and *traJ*.

To assess the impact of *traJ*, we introduced a copy of the gene into pRASK-SP44 and pHL734 and inserted a second copy into pCER (Fig. 1). In brief, the *oriT-traJ* fragment was excised from pUC57-*oriT-traJ* (Fig. 1a) using appropriate restriction enzymes and cloned into the target plasmids, yielding pRASK-SP44 and pHL734 carrying *traJ*, and pCER containing two *traJ* copies (Fig. 1b–d) (see Methods for details).

### The traJ gene increases the number of transconjugants by one order of magnitude in pRASK-SP44 and by two orders of magnitude in pHL734

**Fig. 2:**
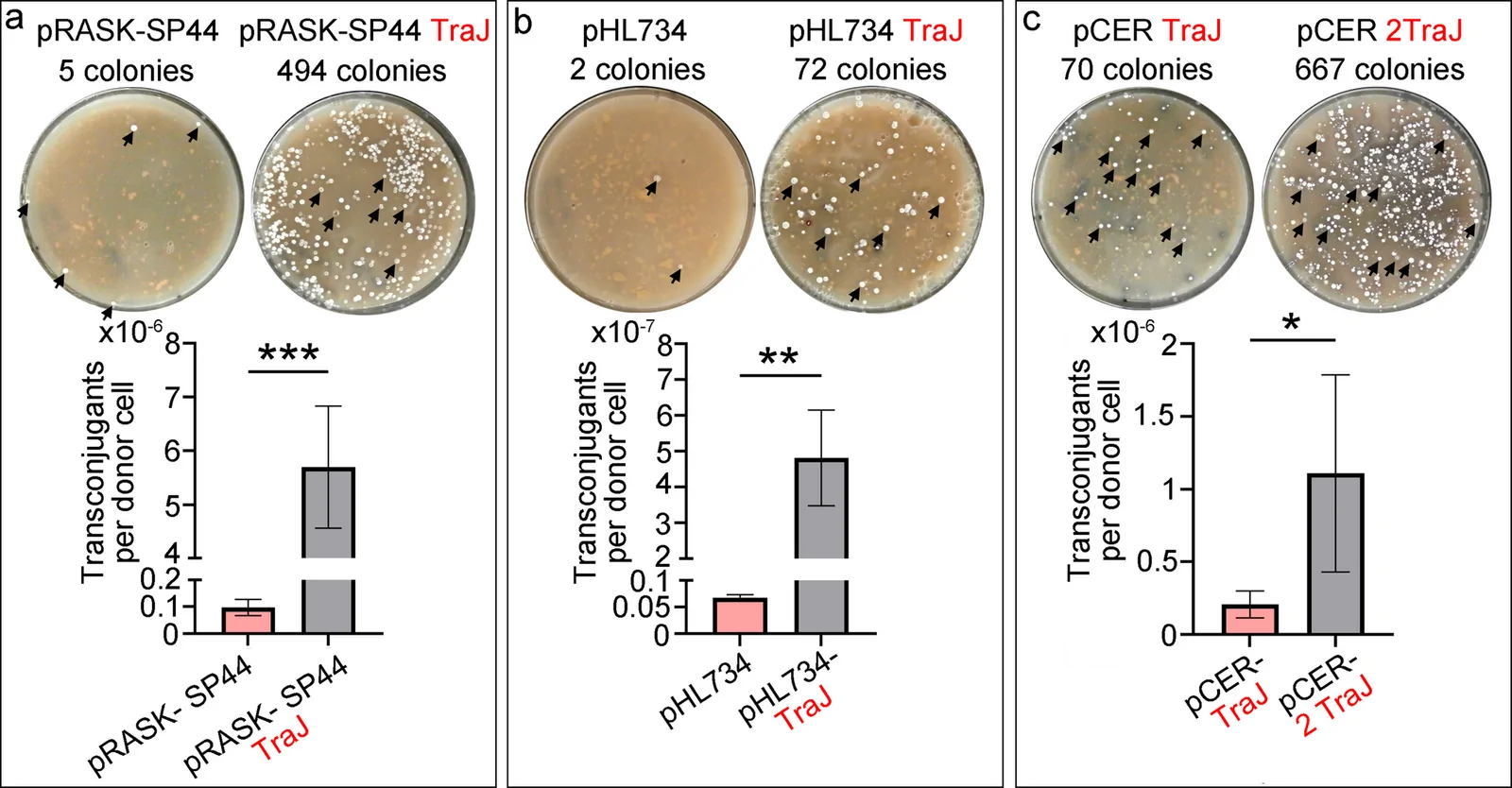
Number of transconjugants per donor cell obtained with pRASK-SP44 and pHL734 plasmids with and without *traJ*, and with pCER carrying one or two copies of *traJ*. **a** pRASK-SP44. **b** pHL734. **c** pCER. Upper panels: macroscopic view of transconjugant colonies on plates inoculated with equal numbers of *S. coelicolor* spores and *E. coli* ET12567 donor cells. Lower panels: quantification of transconjugants per donor cell. Arrows indicate all transconjugant colonies in the low-efficiency conjugations, and a subset of colonies in the high-efficiency conjugations. Asterisks indicate statistically significant differences compared to the plasmid lacking *traJ* (pRASK-SP44 and pHL734) or carrying only one copy of *traJ* (pCER): *p < 0.05, **p < 0.01, ***p < 0.001. Error bars represent standard deviations. Uncropped versions of the gels are shown in Supplementary Fig. 1

**Fig. 3:**
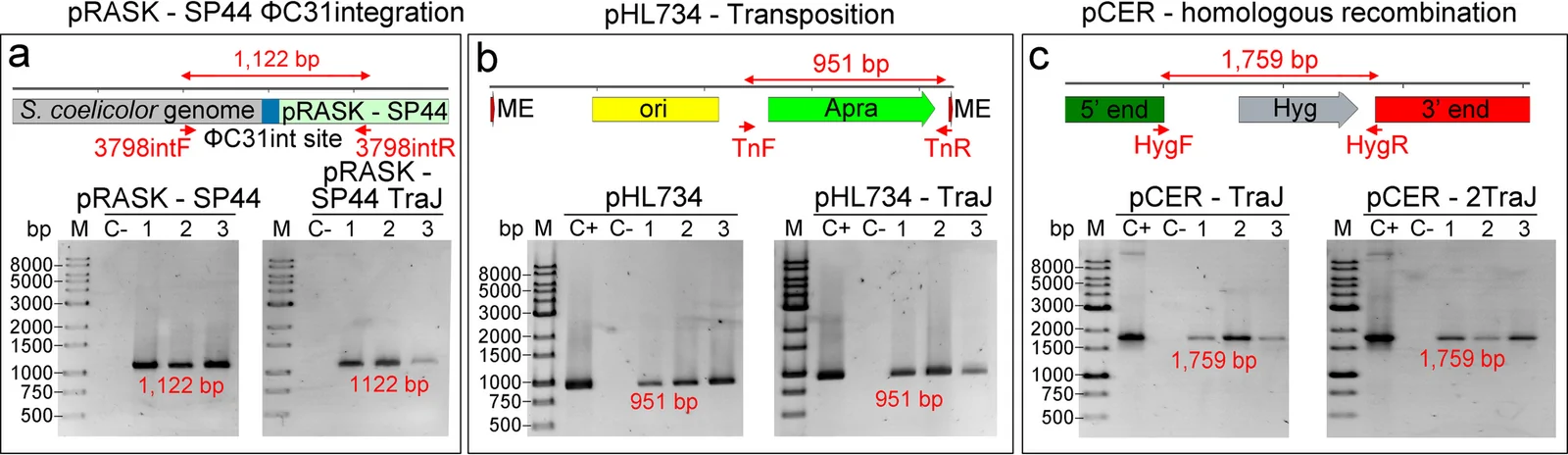
Confirmation of plasmid-derived DNA in the chromosomal DNA of transconjugants. **a** Detection of pRASK integration at the ΦC31 *attB* site. **b** Detection of the apramycin resistance gene in transconjugants carrying the Tn5 transposon delivered by pHL734. **c** Detection of the hygromycin resistance gene in transconjugants in which pCER was incorporated into the chromosome via homologous recombination. Upper panels: schematic representation of the DNA regions amplified by PCR, including primer positions and expected amplicon sizes. Lower panels: agarose gel electrophoresis showing the PCR products. For each experiment, three transconjugants were randomly selected for analysis. C + : positive control (pHL734 or pCER plasmid DNA used as template). C − : negative control (chromosomal DNA from wild-type *S. coelicolor*). M: molecular weight marker

As detailed in the Methods, conjugation experiments were carried out using *E. coli* ET12567/pUZ8002 as the donor strain, which provides the broad-host-range transfer machinery of plasmid RP4, including the *traJ* gene, and *Streptomyces coelicolor* as the recipient model strain. The number of *E. coli* donor cells and *S. coelicolor* recipient spores were quantified and adjusted to ensure consistent conjugation conditions across all experiments (see Methods). The number of antibiotic-resistant transconjugants was normalised to the number of donor cells. The presence of *traJ* led to a tenfold increase in the number of transconjugants per donor cell for pRASK-SP44 (from 1 × 10^–7^ ± 0.3 × 10^–7^ to 5.7 × 10^–6^ ± 1.1 × 10^–6^) (Fig. 2a) and a one–100-fold increase in pHL734 (from 6.7 × 10^–9^ ± 0.5 × 10^–9^ to 4.8 × 10^–7^ ± 1.3 × 10^–7^) (Fig. 2b, the raw colony counts in Supplementary Table 1). To further validate the transconjugants, in addition to antibiotic resistance, three transconjugants were randomly selected for each experiment, and the integration of ΦC31 pRASK-SP44 and insertion of the mini-Tn5 transposon carried by pHL734 were confirmed by PCR (Fig. 3a-b; uncropped versions of the gels are shown in Supplementary Fig. 1).

### A second copy of traJ increases fivefold the number of transconjugants obtained with the pCER plasmid

Incorporating an additional copy of *traJ* into the pCER plasmid increased fivefold transconjugants per donor cell compared to the original pCER plasmid carrying a single *traJ* copy (from 0.21 × 10^–6^ ± 0.1 × 10^–6^ to 1.1 × 10^–6^ ± 0.6 × 10^–6^) (Fig. 2c, the raw colony counts in Supplementary Table 1). As with the other plasmids, insertion of the pCER plasmid into the chromosome of the transconjugants was confirmed by PCR (Fig. 3c; uncropped versions of the gels are shown in Supplementary Fig. 1).

## Discussion

Although conjugation from *E. coli* ET12567 into *Streptomyces* is widely used (Flett et al. 1997; Nikodinovic et al. 2003) and numerous RK2-derived vectors carrying *oriT* have been developed (Bierman et al. 1992; Kieser 2000), a universally effective protocol across *Streptomyces* species has yet to be established (Song et al. 2019; Sun et al. 2014). Conjugation efficiency depends on multiple factors, including spore or mycelial density, growth conditions, donor and recipient pre-treatment, medium composition—particularly ion concentrations—and the selection regime used to distinguish donor and recipient populations (Han et al. 2025; Musiol-Kroll et al. 2019). Even in well-characterised model strains, certain plasmids fail to transfer or do so at very low frequencies for reasons not yet understood (Gomez-Escribano et al. 2025), underscoring the need to better define the molecular determinants of conjugation success.

The presence of *oriT* is the only strict requirement for plasmid mobilization from *E. coli* ET12567, yet additional transfer components may enhance efficiency. TraJ, an essential relaxosome component (Pansegrau et al. 1988) located downstream of *oriT* in RK2 (Larcombe et al. 2024), is often co-cloned with *oriT* in RK2-derived vectors, although some contain *oriT* alone. The main objective of this study was to test whether plasmids carrying *traJ* transfer more efficiently than those lacking it. To this end, *traJ* was introduced into two representative vectors: pHL734, a non-replicative mini-Tn5 transposon vector (Xu et al. 2017), and pRASK-SP44, a ΦC31 integrative plasmid (Fernández-García et al. 2024). We also examined the effect of *traJ* copy number using pCER, developed in our laboratory for chromosomal-end deletion (unpublished). The presence of *traJ* increased transconjugant numbers by one and two orders of magnitude in pRASK-SP44 and pHL734, respectively (Fig. 2a, b), while an additional copy in pCER yielded a fivefold increase (Fig. 2c).

Earlier work by Guiney and Yakobson (1983) identified the *oriT* region of RK2 within a 112 bp segment but showed that a larger 760 bp fragment increased conjugation between *E. coli* strains by five orders of magnitude compared with minimal *oriT*. Although the responsible sequences were not identified, the proximity of *traJ* suggests it may have contributed to this effect. Similarly, Ye et al. (2020) proposed that adding *traJ* to pCRISPomyces-2 might enhance conjugation, though their experiments produced no transconjugants with CRISPR–Cas9 delivery, irrespective of *traJ* presence. In our study, the differing magnitudes of enhancement between pRASK-SP44 and pHL734 (one vs. two orders) likely reflect plasmid-specific mechanisms by which each plasmid integrates or maintains the transferred DNA once inside the recipient cell, ΦC31 site-specific recombination for pRASK-SP44 versus Tn5 transposition for pHL734, which differ in efficiency and stability. Moreover, we established a calibration curve for each *E. coli* ET12567/pUZ8002 donor strain carrying the mobilizable plasmids using overnight cultures, revealing differences in growth fitness that could influence donor–to–recipient ratios, in addition to the conjugation process itself.

Recently, Cochrane et al. (2022) reported that mutations upstream of *traJ* in the RP4-mobilizable PTA-Mob 2.0 plasmid increased conjugation efficiency from *E. coli* to yeast, whereas *traJ* ORF inactivation markedly reduced it. The authors suggested altered promoter activity could reduce *traJ* transcription, although expression levels were not quantified, leaving open the possibility of overexpression. Their observation that *traJ* inactivation diminishes conjugation aligns with our findings in *Streptomyces*. Further work will be required to determine whether their enhanced transfer resulted from elevated or reduced *traJ* expression.

The aim of this work is primarily applied, demonstrating that conjugation from *E. coli* to *Streptomyces* is markedly enhanced by the incorporation of *traJ* into mobilizable plasmids. Further work will be required to elucidate the underlying molecular mechanisms and to apply this knowledge to optimise plasmid DNA delivery across the genus. The positive effect of *traJ* likely reflects increased expression. TraJ acts as a positive regulator of transfer by promoting relaxase recruitment and binding to the 19-bp inverted repeat sequence in the *oriT* (Pansegrau et al. 1990), initiating relaxosome assembly. In our system, TraJ encoded by the mobilizable plasmid, together with that from the pUZ8002 helper plasmid, probably stabilises relaxosome activity by enhancing interactions between the relaxase and auxiliary transfer proteins. This would increase nicking and strand displacement at *oriT*, producing more transfer-competent plasmids and explaining the observed improvements. This hypothesis could be tested in future experiments aimed at quantifying *traJ* expression by qRT-PCR and TraJ protein abundance by Western blotting or quantitative proteomics. If confirmed, conjugation efficiency might be optimised by modulating *traJ* expression via strong or inducible promoters. A secondary explanation, that the *traJ–oriT* region acts as a cis-acting element, probably because that region contains an uncharacterized regulatory element, appears less likely, as *oriT* is the only known DNA site interacting directly with the relaxosome (Pansegrau et al. 1990). Here, we incorporated *traJ* adjacent to *oriT* to preserve the native upstream *traJ* region from RP4, including its promoter and ribosome-binding site. Although *traJ* clearly enhances conjugation efficiency in *S. coelicolor*, yielding frequencies of 5.7 × 10⁻⁶ and 4.8 × 10⁻⁷ transconjugants per donor cell for pRASK-SP44 and pHL734, respectively (Fig. 2), these values remain below those reported for other *Streptomyces* species. Zhang et al. (2019) achieved up to 6.2 × 10⁻^5^ per donor cell in *S. kanamyceticus* using mycelial recipients, while Song et al. (2019) reported 10⁻^5^–10⁻^4^ per donor cell in *S. rimosus* using spores. Although neither study examined *traJ* specifically, both employed pSET152-derived plasmids that naturally contain *traJ* alongside *oriT*. These results emphasise the complexity of conjugation and the influence of strain- and condition-specific factors. Our findings indicate that *traJ* is one such determinant, at least in *S. coelicolor*. Conversely, Du et al. (2012) used pIJ773-derived plasmids containing *oriT* but lacking *traJ* (Gust et al. 2003) and obtained 1.1 × 10⁻^4^ transconjugants per donor cell in *S. lincolnensis*. Testing whether *traJ* can improve transfer in *S. lincolnensis* and other *Streptomyces* species would be informative. Constructs enabling *traJ* overexpression under strong promoters may yield further gains.

In summary, this study reveals that *traJ* is a key determinant of conjugation efficiency from *E. coli* to *Streptomyces*. Its inclusion, and potentially its dosage, can substantially improve plasmid transfer and enable more robust genetic manipulation strategies. While previous studies have mainly focused on optimizing physical or chemical parameters such as media composition, donor-to-recipient ratios, or incubation conditions, our results demonstrate that plasmid architecture, particularly the presence and copy number of *traJ*, is equally critical. Given the widespread use of IncP-type plasmids for conjugation in Gram-positive bacteria, these findings have broader implications for developing genetic tools for genome engineering and natural product discovery in *Streptomyces*.

## Supplementary Information

Below is the link to the electronic supplementary material.

ESM 1 (PDF 387 KB)

ESM 2 (XLSX 10.6 KB)

## Data Availability

The authors declare that the data supporting the findings of this study is available within the article.
