# Supplementary material for: Enhancing conjugation from E. coli to Streptomyces coelicolor by incorporating traJ into mobilizable plasmids
Source: Appl Microbiol Biotechnol. 2025 Dec 9;109(1):262. doi: 10.1007/s00253-025-13662-4 (PMC12696120; doi:10.1007/s00253-025-13662-4)
Supplement: Supplementary file 1 — (PDF 387 KB) [file 253_2025_13662_MOESM1_ESM.pdf]

Supplementary Figure 1. Uncropped versions of the gels shown in Figure 3.

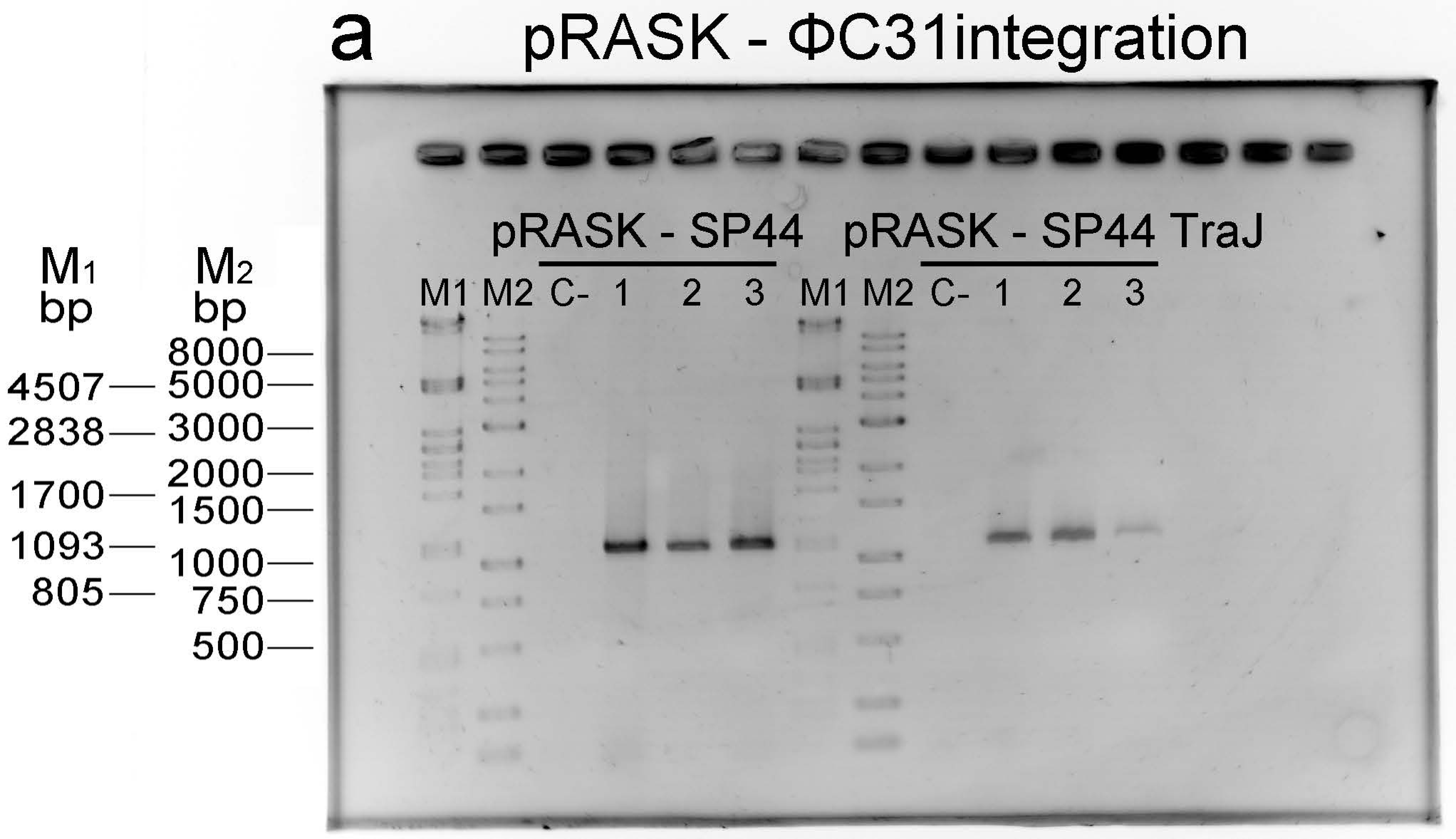

a
pRASK - ΦC31integration
pRASK - SP44
pRASK - SP44 TraJ
M1 M2 C- 1 2 3 M1 M2 C- 1 2 3
M1 bp
M2 bp
4507
2838
1700
1093
805
8000
5000
3000
2000
1500
1000
750
500

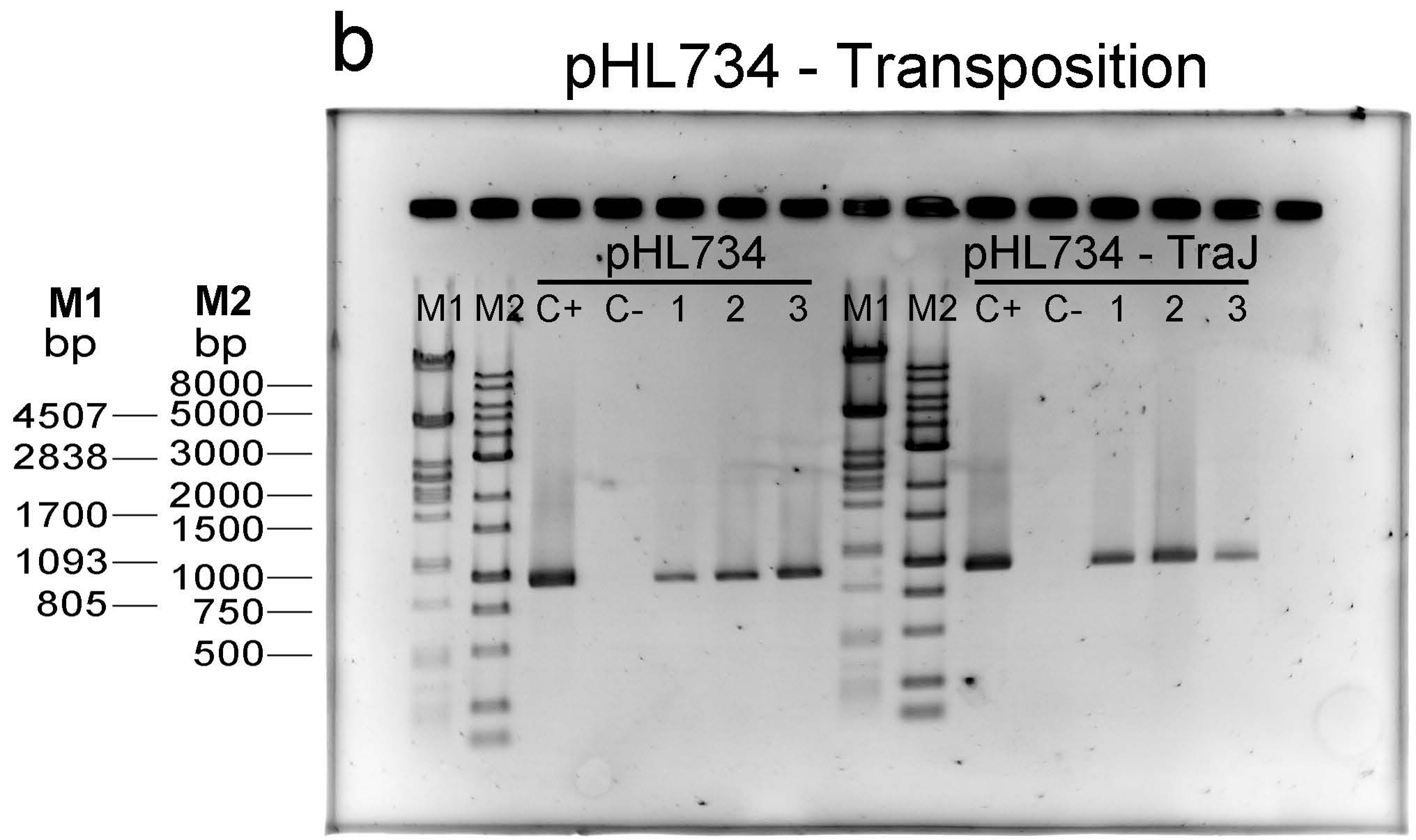
b
pHL734 - Transposition
pHL734
pHL734 - TraJ
M1 M2 C+ C- 1 2 3 M1 M2 C+ C- 1 2 3
M1 bp
M2 bp
8000
4507
5000
2838
3000
2000
1700
1500
1093
1000
805
750
500

C

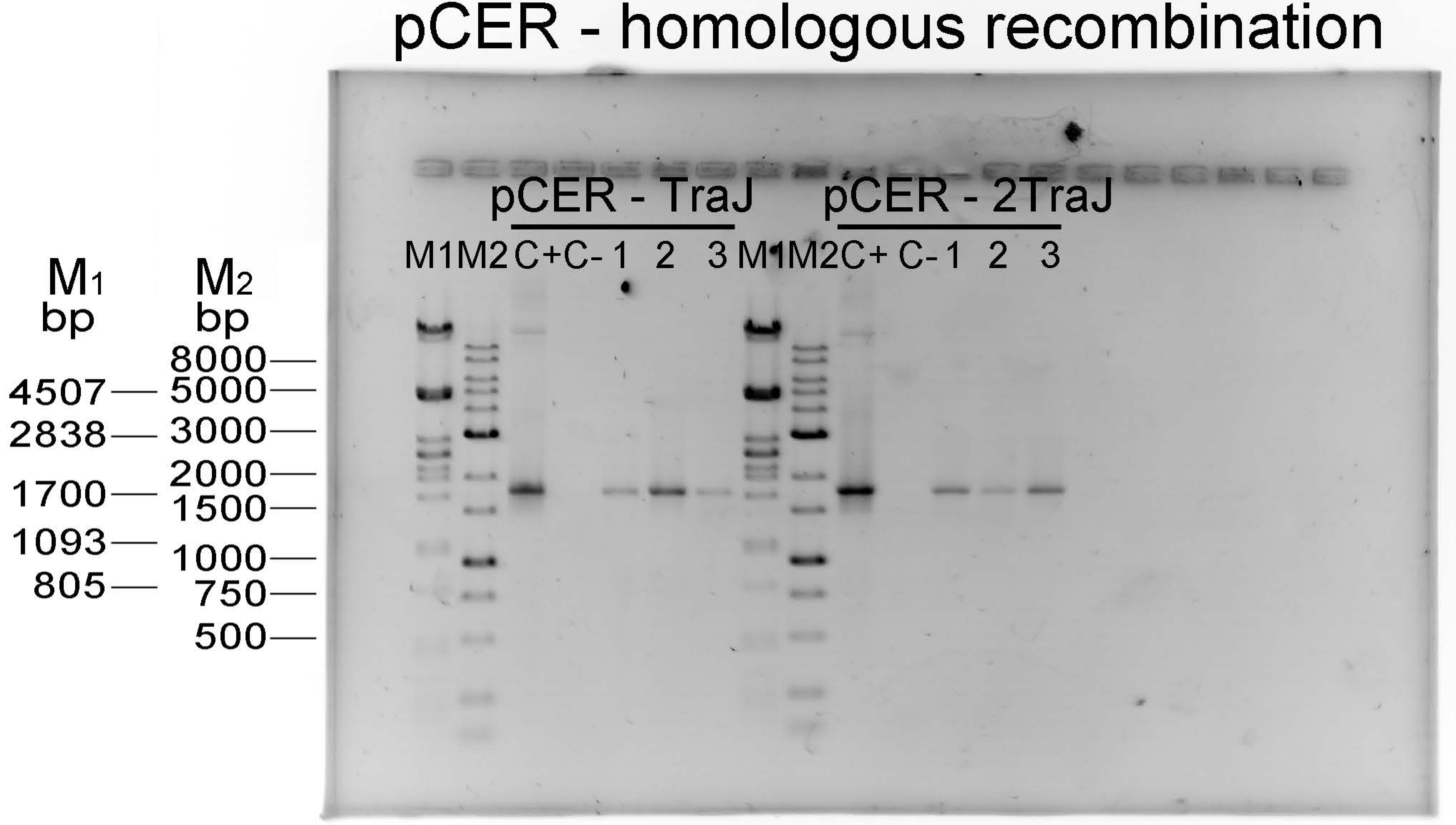
pCER - homologous recombination
pCER - TraJ
pCER - 2TraJ
M1 M2 C+ C- 1 2 3 M1 M2 C+ C- 1 2 3
M1 bp
M2 bp
4507
2838
1700
1093
805
8000
5000
3000
2000
1500
1000
750
500
